# The mediating effect of resilience on pregnancy stress and prenatal anxiety in pregnant women

**DOI:** 10.3389/fpsyt.2022.961689

**Published:** 2022-10-14

**Authors:** Xiabidan Tuxunjiang, Ling Li, Gulijianati Wumaier, Wei Zhang, Bahedana Sailike, Ting Jiang

**Affiliations:** ^1^Department of Public Health, Xinjiang Medical University, Urumqi, China; ^2^Obstetrics Department, The First Affiliated Hospital of Xinjiang Medical University, Urumqi, China

**Keywords:** pregnant women, pregnancy stress, prenatal anxiety, mental resilience, the mediating effect

## Abstract

**Objective:**

To investigate the relationship between pregnancy stress and prenatal anxiety in pregnant women in Urumqi, Xinjiang, and the mediating effect of mental resilience level on the relationship between pregnancy stress and prenatal anxiety.

**Method:**

The investigation involved 750 pregnant women at a tertiary hospital in Urumqi, and included a questionnaire eliciting general demographic information, a pregnancy stress scale (Pregnancy Pressure Scale, PPS), generalized anxiety disorder scale (Generalized Anxiety Disorder-7, GAD-7), and a mental resilience scale (Connor—Davidson resilience scale, CD-RISC). The Bootstrap mediation effect test was used to test the effect relationship between variables, and Amos was used to establish the structural equation model.

**Results:**

Among the 750 participants, 122 (16.2%) had moderate or greater pregnancy stress (PPS > 1), 372 (49.6%) had mild or greater anxiety symptoms (GAD-7 > 5), and 241 (32.1%) had good or higher mental resilience score. Pregnancy stress negatively affected resilience (β = −0.37, *p* < 0.01), and resilience also negatively affected prenatal anxiety (β = −0.12, *p* < 0.01). The mediating effect value of resilience was 8.3%.

**Conclusion:**

Pregnancy stress, mental resilience, and prenatal anxiety were significantly correlated, and mental resilience played a partial mediating role in the influence of pregnancy stress on prenatal anxiety. It is recommended that pregnant women exercise their mental resilience to reduce the incidence of prenatal anxiety and promote physical and mental health.

## Introduction

Anxiety disorder during pregnancy mainly refers to pregnant women during pregnancy feel panic, tension as the main feature of mental health disorders (1, 2). With the emergence of adverse emotions in pregnant women, other negative psychological aspects associated with pregnancy also appear, such as pregnancy pressure. Pregnancy pressure refers to the pressure that pregnant women feel from the environment and work during pregnancy, and it is an inducing factor that aggravates anxiety and depression in pregnant women (3, 4). In recent years, many researchers have paid attention to the mental health of pregnant women, especially anxiety and depression. Studies have shown that excessive pregnancy pressure had a negative impact on the health of pregnant women and the growth of their offspring (5–7). One study showed that low anxiety and cortisol levels and reduced work-life conflicts could prevent fetal complications (6, 7). Besides, research indicated that serious psychological problems during pregnancy can make pregnant women suicidal (8). The response to the new coronavirus in recent years has increased public panic and anxiety, affecting people's mental health (9). Many studies focus on epidemiological investigation and infection prevention measures, but pay little attention to mental health problems (10, 11).

Pregnant womens' ability to self-regulate and adapt well by using their own positive qualities and external environmental resources are called psychological resilience (12, 13). The psychological resilience scale includes 25 self-reported questions and covers five factors: personal competence; high standards and tenacity; trust in one's instincts, tolerance of negative effect, and strengthening effects of stress; positive acceptance of change and secure relationships; control; and spiritual influences (14, 15). Studies have shown that if pregnant women have a high level of resilience they can actively face negative situations and various maladaptive behaviors during pregnancy (16–18). In this study, 750 pregnant women were investigated to study the correlations between pregnancy stress, resilience and prenatal anxiety, and to explore the mediating effect of resilience between pregnancy stress and prenatal anxiety.

## Methods

### Study participants

**Figure 1:**
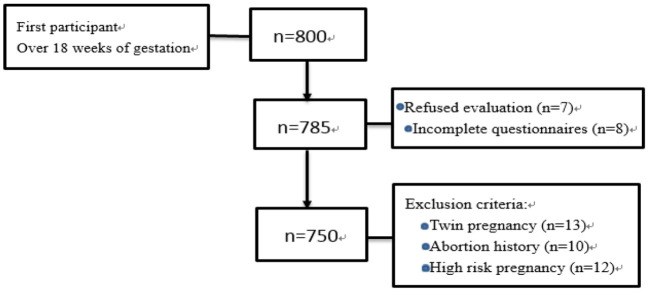
Flow diagram of the narrative review.

This study was carried out in the Department of Obstetrics and Gynecology of A tertiary hospital in Urumqi city, starting from December 2020 to May 2021. The study participants come from districts of Xinjiang, and they were pregnant women who underwent labor examination and we used random sampling methods to randomly distribute questionnaires to the women who came for a prenatal examination on the same day, so as to ensure that the probability of each pregnant woman being selected was equal. Inclusion criteria: clear diagnosis of pregnancy, informed consent, gestational weeks over 18. Exclusion criteria: no informed consent, incomplete data collection, twin pregnancy, have a history of abortion, severe mental disorders, cognitive disorders, hearing disorders, language communication disorders, severe pregnancy complications, and high risk pregnancies. The investigators conducted on-site questionnaire surveys for those women who met the inclusion and exclusion criteria, and the questionnaires were completed independently. If a questionnaire was found to be incomplete, it was not used. We invited 800 women to join this research, and exclude 50 people who do not meet the inclusion criteria. Finally, 750 valid questionnaires were collected, with an effective success rate of 93.7%, as shown in Figure 1.

General demographic questionnaires included general demographic data such as maternal age, educational level, monthly family income, place of residence, and occupation, as well as maternal family environment and living conditions, marital relations, exercise, psychological preparation for pregnancy, and pregnancy knowledge.

### Pregnancy pressure scale

This scale contains three dimensions with a total of 30 entries. Dimension 1 “parent role” contains 15 items, and was the scale of the top 15 entries; dimension 2 “mother and child health and safety” contained eight items, and scaled 16–23; dimension 3 “body shape and physical activity change” contained four entries, namely items 24–27 in the inventory. There were also three items not included in the dimensions: the last three items of the scale (19). The Likert 4 grade scoring method was adopted foe this scale, and the score is the actual total score of the scale divided by the total items of the scale, where 0 points represents no pressure, 0.001–1 points represents mild pressure, 1.001–2 points represents moderate pressure and 2.001–3 points represents severe pressure (3). The higher the score, the greater the pregnancy pressure for a women, and the Cronbach's α coefficient was 0.953.

### Generalized anxiety disorder-7

The Generalized Anxiety Scale, which consist of seven items, used a 4-level scoring system. Each part was scored on a scale of 0–3, with an overall score of 0–4 representing normal, 5–9 representing mild anxiety, 10–14 representing moderate anxiety, and 15–21 representing severe anxiety (20). Out of a total of 21 points, the higher the score, the higher the anxiety level; the Cronbach's α coefficient was 0.885.

### Connor-Davidson resilience scale

This scale includes 3 dimensions of tenacity (13 items), strength (8 items) and optimism (4 items), with a total of 25 items (21). The Likert 5 score is used, and 0–4 points indicate almost never, rarely, sometimes, often and almost always. The total score of the scale was 0–100 points. A score of < 60 indicates poor resilience, 60–70 indicates normal resilience, a score of 70–80 indicates good resilience, and ≥80 indicates excellent resilience (22). The Cronbach's α coefficient is 0.972.

### Statistical methods

The data from the questionnaire were collected, and the information was input into Statistical Package for Social Sciences (SPSS) for Windows version 25.0 for analysis, the enumeration data were expressed as the number of cases and percentage, and the measurement data that obeyed the normal distribution were statistically described. Normally distributed data between groups were analyzed using an independent sample *t*-test or analysis of ANOVA. Pregnancy stress, prenatal anxiety and resilience scores were in line with a normal distribution using Shapiro-Wilk Test (*P* > 0.05). The study also measured the corresponding Cronbach's αs. The mediating effect of resilience between pregnancy stress and prenatal anxiety was tested by non-parametric percentile Bootstrap method (23), the non-parametric percentile method of deviation correction, and *N* = 5,000. Mediation effect analysis proposes to determine whether a mediation effect exists and the type of mediation's corresponding parameters. Parameter refers to influence effect value. When the Bootstrap 95% confidence interval does not include 0, the mediating effect is considered to be statistically significant (24, 25). A mediating effect path analysis chart was produced using Amos23.0 modeling and analysis.

## Results

### General demographic data

Among the 750 pregnant women who met the criteria and were included in this study, 613 (81.7%) were of Han nationality and 137 (18.3%) were of ethnic minorities. Sixty-four (8.5%) were younger than 25-years-old, 601 (80.1%) were between 25- and 35-years-old, and 85 (11.3%) were older than 35-years-old. Among them, 62 (8.3%) had less than high school education, 206 (27.5%) had high school education, 357 (47.6%) had a bachelor's degree, 115 (15.3%) had a master's degree or above, and 10 (1.3%) had others degree. In total, 573 (76.4%) were employed and 177 (23.6%) were housewives; 33 (4.4%) live in rural areas, and 717 (95.6%) live in urban areas.

### Pregnancy stress, prenatal anxiety, and mental resilience level

The data were analyzed statistically using SPSS 25.0. In this study, the prenatal anxiety score of the pregnant women was (4.98 ± 3.38), the pregnancy stress score was (0.60 ± 0.48), and its' three dimension scores are (0.18 ± 0.21; 0.24 ± 0.17; 0.11 ± 0.10); the mental resilience score was (60.17 ± 19.44), and its' three dimension scores are (30.31 ± 10.47; 20.36 ± 6.39; 9.50 ± 3.46); In this study, 122 participants (16.2%) had moderate or greater pregnancy stress (PPS>1), 372 (49.6%) had mild or greater anxiety symptoms (GAD-7>5), and 241 (32.1%) had a good or higher mental resilience score of 70. There were statistically significant differences in the score of the GAD-7 scale among pregnant women with different educational levels and monthly family incomes, and statistically significant differences in the score for the PPS scale among pregnant women with different ages, monthly family incomes, and occupations. Except for ethnicity resilience scores were statistically significant in different demographic data, as shown in Table 1.

**Table 1:** GAD-7, PPS, and CD-RISC scores of pregnant women with different demographic characteristics.

**Variable**	***N* (%)**	**GAD-7**	**PPS**	**CD-RISC**
**Nationality**				
Ethnic Han	613 (81.7%)	4.93 ± 3.93	0.59 ± 0.48	60.29 ± 19.52
Minority	137 (18.3%)	5.20 ± 3.39	0.66 ± 0.50	59.64 ± 19.18
*t*		−0.729	−1.47	0.335
*p*		0.446	0.143	0.723
**Age**				
≤ 25	64 (8.5%)	6.39 ± 4.01	0.77 ± 0.60	47.97 ± 20.33
25–35	601 (80.1%)	4.87 ± 3.89	0.59 ± 0.47	60.77 ± 18.96
≥35	85 (11.3%)	4.72 ± 2.99	0.54 ± 0.43	65.09 ± 18.76
*F*		1.294	4.903	6.271
*p*		0.170	0.008	0.028
**Education**				
Lower than high school	62 (8.3%)	5.21 ± 3.92	0.59 ± 0.57	53.00 ± 21.08
High school or technical secondary	206 (27.5%)	5.31 ± 3.97	0.59 ± 0.49	55.60 ± 20.73
Bachelor	357 (47.6%)	4.83 ± 3.83	0.60 ± 0.47	62.77 ± 17.63
Master	115 (15.3%)	4.71 ± 3.56	0.62 ± 0.43	65.52 ± 18.32
Others	10 (1.3%)	5.40 ± 3.53	0.87 ± 0.23	44.30 ± 19.52
*F*		2.204	0.882	10.937
*p*		0.002	0.474	0.000
**Monthly family income**				
≤ 3,000	40 (5.3%)	6.98 ± 4.83	0.75 ± 0.65	51.68 ± 22.51
3,001–5,000	194 (25.9%)	5.53 ± 3.99	0.67 ± 0.54	54.79 ± 20.61
5,001–8,000	218 (29.1%)	4.70 ± 3.69	0.60 ± 0.44	59.45 ± 19.09
>8,000	298 (39.7%)	4.57 ± 3.57	0.54 ± 0.44	65.33 ± 16.99
*F*		1.885	4.109	15.442
*p*		0.010	0.007	0.000
**Occupation**				
Full-Time job	573 (76.4%)	4.77 ± 3.69	0.58 ± 0.46	62.05 ± 18.59
Housewives	177 (23.6%)	5.65 ± 4.21	0.67 ± 0.55	54.08 ± 20.94
*t*		1.003	−2.1	4.832
*p*		0.316	0.036	0.000
**Living site**				
Village	33 (4.4%)	5.61 ± 4.81	0.68 ± 0.65	45.18 ± 22.09
City	717 (95.6%)	4.95 ± 3.78	0.60 ± 0.48	60.86 ± 19.05
*t*		0.958	0.979	−4.588
*p*		0.339	0.328	0.000

### Correlation analysis of pregnancy stress, resilience, and prenatal anxiety

The data of this study conform to normality by Shapiro-Wilk Test (*p* > 0.05). According to Pearson's correlation analysis, prenatal anxiety was significantly positively correlated with pregnancy stress (*p* < 0.01), resilience was significantly negatively correlated with prenatal anxiety (*p* < 0.01), and resilience was significantly negatively correlated with pregnancy stress (*p* < 0.01), as shown in Table 2.

**Table 2:** Correlation analysis of prenatal anxiety, pregnancy stress, and resilience.

**Variable**	**Prenatal anxiety**	**Pregnancy stress**	**Resilience**
Prenatal anxiety	1	–	–
Pregnancy stress	0.596**	1	–
Resilience	−0.323**	−0.340**	1

**At a 0.01 level (double tails), the correlation was significant.

### The mediating effect of resilience between pregnancy stress and prenatal anxiety

Using the Bootstrap mediating effect test, this study assumes that resilience plays a mediating role between pregnancy stress and prenatal anxiety. After the analysis of the mediating effect, it was found that the total effect and indirect effect of pregnancy stress on prenatal anxiety were significant (*p* < 0.01). The confidence interval of the mediating effect of resilience as a mediating variable between pregnancy stress and prenatal anxiety was [0.024–0.074], as shown in Table 3.

**Table 3:** Bootstrap analysis of the mediating effect of resilience between pregnancy stress and prenatal anxiety.

**Path**	**(Effect)**	**(SE)**	**95% (CI)**	**Effect value (%)**
a: Pressure → resilience	−0.34	0.034	−0.408 ~−0.273	–
b: Resilience → anxiety	−0.14	0.031	−0.196 ~−0.075	–
c': Pressure → anxiety (direct effect)	0.55	0.031	0.490 ~ 0.611	91.7
c: Pressure → anxiety (total effect)	0.60	0.029	0.539 ~ 0.654	100
Pressure → resilience → anxiety (indirect effect)	0.05	0.012	0.024 ~ 0.074	8.3

**Figure 2:**
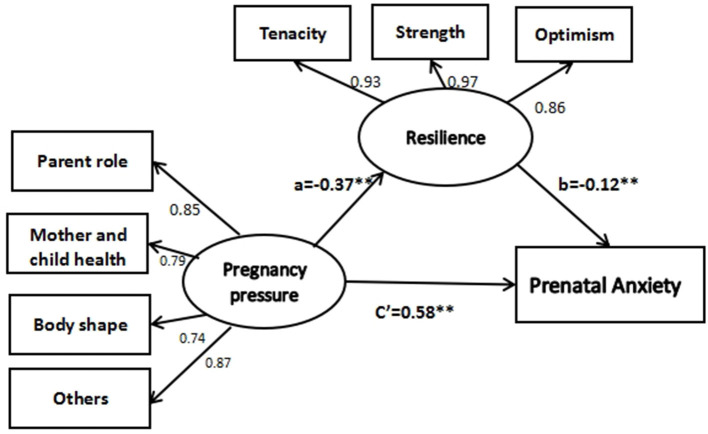
The mediating effect path diagram showing the mediating variable resilience.

With resilience as the mediating variable, pregnancy pressure as the independent variable, and prenatal anxiety as the dependent variable, Amos was introduced to develop a result equation model. The results showed that the path coefficients of the model were statistically significant (*p* < 0.01). The fitting indexes of the structural equation model were as follows: CMIN/DF = 4.042, RMSEA = 0.064, GFI = 0.977, AGFI = 0.954, CFI = 0.987, and TLI = 0.980. The results showed that pregnancy pressure negatively affected resilience (β = −0.37, *p* < 0.01), and resilience negatively affected prenatal anxiety (β = −0.12, *p* < 0.01). According to the path analysis chart, the intermediary effect a^*^b = c–c', namely −0.37 × −0.12 = 0.60–0.55, indicating that the intermediary effect is exists (see Figure 2). The mediating effect (a^*^b = 0.05) accounted for 8.3% of the total effect (0.60), indicating that resilience partially mediated the relationship between pregnancy stress and prenatal anxiety.

## Discussion

In this study, it was found that the overall pregnancy stress level in pregnant women was below the level of mild stress, and the score for the stressors (mother–child health and safety) was the highest. It appeared that worrying about mother-child health was the main stressor, which was inconsistent with the results of a previous study that the main stressor was the stress caused by the parenting role (26). The analysis of the reasons for this difference showed that the gestational age of the sample studied by Guo et al. was ≥37 weeks, while the pregnant women in our study had no limit on gestational age. The closer to the birth, the more fearful the pregnant woman is about changing her role (19, 27). Most of the pregnant women in our group had mild prenatal anxiety. The prenatal anxiety score was positively correlated with family monthly income, which was consistent with the previous research showing that the higher the family monthly income, the lower the prenatal anxiety level (28). Less than half of the pregnant women were at high or higher levels of resilience. Resilience is negatively correlated with maternal prenatal anxiety, indicating that maternal resilience can alleviate maternal prenatal anxiety (29, 30).

Pregnancy pressure, prenatal anxiety, and resilience were significantly correlated. Pregnancy pressure was positively correlated with prenatal anxiety, indicating that the greater the pregnancy pressure, the higher the degree of prenatal anxiety, which is consistent with the results of other research (31, 32). Therefore, pregnant women should be given care during pregnancy, which can reduce their pressure and anxiety (33). There was a negative correlation between resilience and prenatal anxiety, indicating that the level of resilience is a protective factor for prenatal anxiety: the higher the level of resilience, the lower the degree of prenatal anxiety, which is consistent with the results of previous studies (34). Pregnant womens' resilience will affect their ability to cope with major life events. The physiological changes caused by pregnancy itself also exert stress, and for pregnant women who cannot follow positive strategies to manage their emotions effectively and adapt to the state of pregnancy, it is difficult to maintain mental health, thus affecting their overall health (35, 36).

In this study, the score for the resilience optimism dimension of pregnant women was high, and 63.8% of pregnant women exceeded the average score of this dimension. However, the score for the tenacity dimension was lowest, which is consistent with the results of previous studies (37). This result suggests that we should focus on helping pregnant women to improve their resilience (38). This study concluded that the psychological resilience of pregnant women had an intermediary role in regulating pregnancy stress and prenatal anxiety. Emotion can be regulated by psychological resilience (39, 40). Good psychological resilience can produce positive emotions, so that pregnant women can effectively reduce the impact of stress on negative emotions (41, 42).

To summarize, resilience, as an intermediary, can affect both maternal pregnancy stress and maternal prenatal anxiety, as a protective factor against both (43). The higher the level of resilience of pregnant women, the lower the pregnancy pressure and the milder the symptoms of prenatal anxiety (44). In addition, resilience played a partial mediating effect in the relationship between pregnancy stress and prenatal anxiety in pregnant women, that is, pregnancy stress could directly affect prenatal anxiety, or indirectly affect prenatal anxiety through resilience (45, 46).

This study explored the relationship between pregnancy stress and prenatal anxiety in pregnant women, and verified the mediating effect of resilience between them. However, this study had certain limitations. First, the study used a cross-sectional study design, which cannot accurately determine causal relationships, when compared with a cohort study. Second, the study did not limit the gestational age of pregnant women during sample selection, although previous studies have shown that gestational age was one of the important factors affecting prenatal depression and pregnancy pressure (47). Therefore, the influence of gestational age on prenatal depression and pregnancy stress was not examined in this study. Last but not least, this study participants did not exclude pregnant women conceived through assisted reproductive techniques.

## Data availability statement

The raw data supporting the conclusions of this article will be made available by the authors, without undue reservation.

## Ethics statement

The studies involving human participants were reviewed and approved by Ethics Committee of Xinjiang Medical University. The patients/participants provided their written informed consent to participate in this study.

## Author contributions

XT and LL processing data. WZ and GW are participate in writing articles. BS and TJ making tables and figures. All authors contributed to the article and approved the submitted version.

## Funding

This work was supported by the Thirteen Five Key Science—Public Health and Preventive Medicine (Grant No. 11091121204) and the Nature Science Foundation of Xinjiang autonomous region (Grant No. 2018D01C146).

## Conflict of interest

The authors declare that the research was conducted in the absence of any commercial or financial relationships that could be construed as a potential conflict of interest.

## Publisher's note

All claims expressed in this article are solely those of the authors and do not necessarily represent those of their affiliated organizations, or those of the publisher, the editors and the reviewers. Any product that may be evaluated in this article, or claim that may be made by its manufacturer, is not guaranteed or endorsed by the publisher.
